# Supplementary material for: The impact of postoperative glucocorticoids on complications after head and neck cancer surgery with free flap reconstruction: A retrospective study
Source: PLoS One. 2025 Mar 11;20(3):e0319655. doi: 10.1371/journal.pone.0319655 (PMC11896068; doi:10.1371/journal.pone.0319655)
Supplement: S1 Fig — (DOCX) [file pone.0319655.s001.docx]

Screening of patients

(n=1023)

Excluded (n=312)

- Take glucocorticoids (n = 2)
- Immunosuppressive state or taking immunosuppressants (n = 4)
- Take glucocorticoids and immunosuppressants (n=2)
- Head and neck radiotherapy (n = 130)
- Clinical records were incomplete (n =174)

Conclusions drawn

Statistical analysis

High-dose steroid group

(n = 307)

Control group

(n = 404)

Elderly patients meeting

the inclusion criteria (n = 711)
