# Supplementary material for: The impact of postoperative glucocorticoids on complications after head and neck cancer surgery with free flap reconstruction: A retrospective study
Source: PLoS One. 2025 Mar 11;20(3):e0319655. doi: 10.1371/journal.pone.0319655 (PMC11896068; doi:10.1371/journal.pone.0319655)
Supplement: S1 Table — (DOCX) [file pone.0319655.s003.docx]

| Classification of Surgical Complications | |
| --- | --- |
| **Grade** | **Definition** |
| Grade Ⅰ | Any deviation from the normal postoperative course without the need for pharmacological treatment or surgical, endoscopic, and radiological interventions  Allowed therapeutic regimens are: drugs as antiemetics, antipyretics, analgetics, diuretics, electrolytes, and physiotherapy. This grade also includes wound infections opened at the beside |
| Grade Ⅱ | Requiring pharmacological treatment with drugs other than such allowed for grade Ⅰ complications  Blood transfusions and total parenteral nutrition are also included |
| Grade Ⅲ | Requiring surgical, endoscopic or radiological intervention |
| Grade Ⅲa | Intervention not under general anesthesia |
| Grade Ⅲb | Intervention under general anesthesia |
| Grade Ⅳ | Life-threatening complication (including CNS complications)* requiring IC/ICU management |
| Grade Ⅳa | Single organ dysfunction (including dialysis) |
| Grade Ⅳb | Multiorgan dysfunction |
| Grade Ⅴ | Death of a patient |
| Suffix “d” | If the patient suffers from a complication at the time of discharge, the suffix “d” (for “disability”) is added to the respective grade of complication. This label indicates the need for a follow-up to fully evaluate the complication. |
| *Brain hemorrhage, ischemic stroke, subarrachnoidal bleeding, but excluding transient ischemic attacks.  CNS, central nervous system; IC, intermediate care; ICU, intensive care unit. | |
