# Supplementary material for: The impact of postoperative glucocorticoids on complications after head and neck cancer surgery with free flap reconstruction: A retrospective study
Source: PLoS One. 2025 Mar 11;20(3):e0319655. doi: 10.1371/journal.pone.0319655 (PMC11896068; doi:10.1371/journal.pone.0319655)
Supplement: S2 Table — (DOCX) [file pone.0319655.s004.docx]

**S2 Table. Postoperative data and complications.**

| characteristic | Control group  (N=404) | | High-dose steroid group  (N=307) | P value |
| --- | --- | --- | --- | --- |
| **Glycemic control ^a^** |  | |  | **0.0067** |
| Good | 199 (49.3) | | 119 (38.8) |  |
| Bad | 205 (50.7) | | 188 (61.2) |  |
| Pneumonia ^a^ |  | |  | 0.1041 |
| No | 239 (59.2) | | 162 (52.8) |  |
| Yes | 165 (40.8) | | 145 (47.2) |  |
| **Atelectasis ^a^** |  | |  | **0.034** |
| No | 400 (99.0) | | 296 (96.4) |  |
| Yes | 4 (1.0) | | 11 (3.6) |  |
| Pulmonary embolism ^a^ |  | |  | 0.7142 |
| No | 401 (99.3) | | 303 (98.7) |  |
| Yes | 3 (0.7) | | 4 (1.3) |  |
| Respiratory failure ^a^ |  | |  | 0.3998 |
| No | 394 (97.5) | | 303 (98.7) |  |
| Yes | 10 (2.5) | | 4 (1.3) |  |
| Pulmonary complications ^a^ |  | |  | 0.1083 |
| No | 240 (59.4) | | 163 (53.1) |  |
| Yes | 164 (40.6) | | 144 (46.9) |  |
| **Flap infection ^a^** |  | |  | **<0.0001** |
| No | 356 (88.1) | | 180 (58.6) |  |
| Yes | 48 (11.9) | | 127 (41.4) |  |
| Postoperative bleeding ^a^ |  | |  | 0.1895 |
| No | 395 (97.8) | | 294 (95.8) |  |
| Yes | 9 (2.2) | | 13 (4.2) |  |
| **Flap dehiscence** ^a^ |  | |  | **0.0221** |
| No | 394 (97.5) | | 288 (93.8) |  |
| Yes | 10 (2.5) | | 19 (6.2) |  |
| **Seroma/Fistula formation** ^a^ |  | |  | **<0.0001** |
| No | 373 (92.3) | | 222 (72.3) |  |
| Yes | 31 (7.7) | | 85 (27.7) |  |
| **Partial flap necrosis** ^a^ |  | |  | **0.0191** |
| No | 378 (93.6) | | 271 (88.3) |  |
| Yes | 26 (6.4) | | 36 (11.7) |  |
| Flap crisis ^a^ |  | |  | 1 |
| No | 393 (97.3) | | 299 (97.4) |  |
| Yes | 11 (2.7) | | 8 (2.6) |  |
| **Flap complications ^a^** |  | |  | **<0.0001** |
| No | 322 (79.7) | | 144 (46.9) |  |
| Yes | 82 (20.3) | | 163 (53.1) |  |
| Hepatic insufficiency ^a^ |  | |  | 0.422 |
| No | 263 (65.1) | | 190 (61.9) |  |
| Yes | 141 (34.9) | | 117 (38.1) |  |
| Acute pancreatitis ^a^ |  | |  | 0.8111 |
| No | 403 (99.8) | | 305 (99.3) |  |
| Yes | 1 (0.2) | | 2 (0.7) |  |
| Ileus ^a^ |  | |  | 1 |
| No | 403 (99.8) | | 306 (99.7) |  |
| Yes | 1 (0.2) | | 1 (0.3) |  |
| Digestive complications ^a^ |  | |  | 0.2241 |
| No | 260 (64.4) | | 183 (59.6) |  |
| Yes | 144 (35.6) | | 124 (40.4) |  |
| Cardiac insufficiency ^a^ |  | |  | 0.8027 |
| No | 357 (88.4) | | 274 (89.3) |  |
| Yes | 47 (11.6) | | 33 (10.7) |  |
| Cerebral embolism ^a^ |  | |  | 0.0767 |
| No | 402 (99.5) | | 300 (97.7) |  |
| Yes | 2 (0.5) | | 7 (2.3) |  |
| AKI ^a^ |  | |  | 1 |
| No | 399 (98.8) | | 304 (99.0) |  |
| Yes | 5 (1.2) | | 3 (1.0) |  |
| Electrolyte imbalances ^a^ |  | |  | 0.9635 |
| No | 264 (65.3) | | 202 (65.8) |  |
| Yes | 140 (34.7) | | 105 (34.2) |  |
| DVT ^a^ |  | |  | 0.9998 |
| No | 392 (97.0) | | 297 (96.7) |  |
| Yes | 12 (3.0) | | 10 (3.3) |  |
| Postoperative delirium ^a^ |  | |  | 0.4073 |
| No | 357 (88.4) | | 264 (86.0) |  |
| Yes | 47 (11.6) | | 43 (14.0) |  |
| Unplanned surgery ^a^ |  | |  | 0.3171 |
| No | 388 (96.0) | | 289 (94.1) |  |
| Yes | 16 (4.0) | | 18 (5.9) |  |
| Death ^a^ |  | |  | 0.4517 |
| No | 402 (99.5) | | 303 (98.7) |  |
| Yes | 2 (0.5) | | 4 (1.3) |  |
| **Clavien-Dindo classification** ^a^ | |  |  | **0.0034** |
| 1 | 87 (21.5) | | 35 (11.4) |  |
| 2 | 284 (70.3) | | 239 (77.9) |  |
| 3a | 4 (1.0) | | 8 (2.6) |  |
| 3b | 11 (2.7) | | 12 (3.9) |  |
| 4a | 14 (3.5) | | 8 (2.6) |  |
| 4b | 2 (0.5) | | 0 (0.0) |  |
| 5 | 2 (0.5) | | 5 (1.6) |  |
| ICU ^b^ | 2.60 (1.69) | | 2.64 (2.07) | 0.7493 |
| **PHS** ^b^ | 13.33 (3.63) | | 14.98 (4.25) | **<0.0001** |
| **Post-discharge information** | | | | |
| **NPR** ^a^ |  | |  | **<0.0001** |
| No | 354 (87.6) | | 174 (56.7) |  |
| Yes | 50 (12.4) | | 133 (43.3) |  |
| **Difficulty swallowing** ^a^ |  | |  | **0.0001** |
| No | 389 (96.3) | | 271 (88.3) |  |
| Yes | 15 (3.7) | | 36 (11.7) |  |
| **Recurrence** ^a^ |  | |  | **<0.0001** |
| No | 393 (97.3) | | 243 (79.2) |  |
| Yes | 11 (2.7) | | 64 (20.8) |  |
| **Metastasis** ^a^ |  | |  | **<0.0001** |
| No | 391 (96.8) | | 264 (86.0) |  |
| Yes | 13 (3.2) | | 43 (14.0) |  |
| Lung infections ^a^ |  | |  | 0.1267 |
| No | 395 (97.8) | | 293 (95.4) |  |
| Yes | 9 (2.2) | | 14 (4.6) |  |
| Delayed healing ^a^ |  | |  | 0.2811 |
| No | 396 (98.0) | | 296 (96.4) |  |
| Yes | 8 (2.0) | | 11 (3.6) |  |

**a: Data are presented as n (%) for categorial values; b: Data are presented as mean (standard deviation) for continuous variables; AKI: Acute kidney injury; DVT:** **Deep vein thrombosis; PHS:** **Postoperative hospital stay; NPR:** **Non-planned readmissions within the first year.**
