# Supplementary material for: The impact of postoperative glucocorticoids on complications after head and neck cancer surgery with free flap reconstruction: A retrospective study
Source: PLoS One. 2025 Mar 11;20(3):e0319655. doi: 10.1371/journal.pone.0319655 (PMC11896068; doi:10.1371/journal.pone.0319655)
Supplement: S3 Table — (DOCX) [file pone.0319655.s005.docx]

**S3 Table. The relationship between high-dose steroid group and non-planned readmissions in different subgroups compared to the control group.**

| Subgroup | Count | Percent | OR, 95%CI | P value | Interaction P |
| --- | --- | --- | --- | --- | --- |
| **Primary lesion location** | |  |  |  | **0.309** |
| cheek | 173 | 24.3 | 4 (1.83, 8.78) | 0.001 |  |
| tongue | 235 | 33.1 | 7.04 (3.43, 14.45) | <0.001 |  |
| gingiva | 155 | 21.8 | 7.07 (2.82, 17.73) | <0.001 |  |
| others | 148 | 20.8 | 8.63 (3.19, 23.37) | <0.001 |  |
| **Pathological type** | |  |  |  | **0.108** |
| SCC | 644 | 90.6 | 5.49 (3.64, 8.28) | <0.001 |  |
| others | 67 | 9.4 | 22.6 (3.68, 138.82) | 0.001 |  |
| **T stage** | |  |  |  | **0.573** |
| 1 | 62 | 8.7 | 49.07 (2.9, 828.73) | 0.007 |  |
| 2 | 377 | 53 | 5.55 (3.17, 9.7) | <0.001 |  |
| 3 | 108 | 15.2 | 20.98 (6.02, 73.13) | <0.001 |  |
| 4 | 164 | 23.1 | 4.57 (2.03, 10.28) | <0.001 |  |

**SCC:** **Squamous cell carcinoma.**
