# Supplementary material for: The impact of postoperative glucocorticoids on complications after head and neck cancer surgery with free flap reconstruction: A retrospective study
Source: PLoS One. 2025 Mar 11;20(3):e0319655. doi: 10.1371/journal.pone.0319655 (PMC11896068; doi:10.1371/journal.pone.0319655)
Supplement: Attachment — Submitted filename: Response to Reviewers.docx [file pone.0319655.s006.docx]

We thank the Reviewers and Editors for their constructive feedback to improve the manuscript and allow a resubmission to PLOS ONE. We have considered all the comments carefully and inserted our one-to-one responses below. Our responses are in blue font, the modified manuscript text is in red font to improve readability. We believe that the updated manuscript has been substantially improved. Thank you in advance for your continued consideration.

Journal requirements:

When submitting your revision, we need you to address these additional requirements.

1. Please ensure that your manuscript meets PLOS ONE's style requirements, including those for file naming. The PLOS ONE style templates can be found at

https://journals.plos.org/plosone/s/file?id=wjVg/PLOSOne_formatting_sample_main_body.pdf and

[https://journals.plos.org/plosone/s/file?id=ba62/PLOSOne_formatting_sample_title_authors_affiliations.pdf](https://track.editorialmanager.com/CL0/https:%2F%2Fjournals.plos.org%2Fplosone%2Fs%2Ffile%3Fid=ba62%2FPLOSOne_formatting_sample_title_authors_affiliations.pdf/1/010f01945ec693dd-238b815c-c231-49fd-afe0-b139abc7f790-000000/YFpzlbA2NVuLIB9HUgysI69jlWHr0xVYPw5i5Vvnjh8=193)

**Response:**

Thank you for your comment. Based on the template you provided, we have modified the manuscript format and ensured it meets the PLOS ONE requirements.

2. We note that you have indicated that there are restrictions to data sharing for this study. For studies involving human research participant data or other sensitive data, we encourage authors to share de-identified or anonymized data. However, when data cannot be publicly shared for ethical reasons, we allow authors to make their data sets available upon request. For information on unacceptable data access restrictions, please see <http://journals.plos.org/plosone/s/data-availability#loc-unacceptable-data-access-restrictions>.

Before we proceed with your manuscript, please address the following prompts:

a) If there are ethical or legal restrictions on sharing a de-identified data set, please explain them in detail (e.g., data contain potentially identifying or sensitive patient information, data are owned by a third-party organization, etc.) and who has imposed them (e.g., a Research Ethics Committee or Institutional Review Board, etc.). Please also provide contact information for a data access committee, ethics committee, or other institutional body to which data requests may be sent.

b) If there are no restrictions, please upload the minimal anonymized data set necessary to replicate your study findings to a stable, public repository and provide us with the relevant URLs, DOIs, or accession numbers. Please see http://www.bmj.com/content/340/bmj.c181.long for guidelines on how to de-identify and prepare clinical data for publication. For a list of recommended repositories, please see https://journals.plos.org/plosone/s/recommended-repositories. You also have the option of uploading the data as Supporting Information files, but we would recommend depositing data directly to a data repository if possible.

Please update your Data Availability statement in the submission form accordingly.

**Response:**

Thank you for your attention and recognition of our research work. We understand your request for data sharing. However, our data belongs to a third-party organization (i.e., the Ninth People's Hospital Affiliated to Shanghai Jiao Tong University School of Medicine). According to the requirements of the Ethics Committee of the Ninth People's Hospital Affiliated to Shanghai Jiao Tong University School of Medicine, any sharing of patient data requires the approval of the Ethics Committee. However, based on reasonable needs, editors, reviewers, or readers can obtain the original data supporting our research through the Ethics Committee (email: shjyiec@163.com). In addition, we have updated the Data Availability statement in the manuscript: “The data supporting the findings of this study are available from the Ethics Committee of the Ninth People's Hospital Affiliated to Shanghai Jiao Tong University School of Medicine (email: shjyiec@163.com) upon reasonable request.”

3. Please review your reference list to ensure that it is complete and correct. If you have cited papers that have been retracted, please include the rationale for doing so in the manuscript text, or remove these references and replace them with relevant current references. Any changes to the reference list should be mentioned in the rebuttal letter that accompanies your revised manuscript. If you need to cite a retracted article, indicate the article’s retracted status in the References list and also include a citation and full reference for the retraction notice.

**Response:**

Thank you for your kind comments. We reviewed our reference list to confirm that it is complete and correct. None of the articles we cited have been retracted, so no changes have been made to the reference list.

Review Comments to the Author

Please use the space provided to explain your answers to the questions above. You may also include additional comments for the author, including concerns about dual publication, research ethics, or publication ethics. (Please upload your review as an attachment if it exceeds 20,000 characters)

Reviewer #1: 1. The topic is not unique but worthy of researching
2. There are many papers in google scholar and Refseek about this topic since 2020
3. Ethical approval and/or study registration is mentioned. Please clarify it in the method section

**Response:**

Thank you for your comment. As you can see, we have made a detailed statement of ethical approval in the “Materials and methods” section: “Approval was obtained from the center's ethics committee (Ethics Reference: SH9H-2024-T315-1), and, due to its retrospective nature, the requirement for informed consent was waived.”

(The position of the original manuscript: Page 6, Lines 95-97)
4. the title is attractive
5. the running title is not suitable

**Response:**

Thank you for your comment. We have rewritten the running title: “Glucocorticoids and Complications after Head and Neck Cancer Surgery”
6. The abstract is clear. It is too long. You can shorten the background and the conclusion.

**Response:**

Thank you for your valuable comment, which we have carefully considered. As per your suggestion, we have made revisions to the “Abstract” section. Please refer to the following updated version, Page 2-3, Lines 16-19, 37-41 of the revised version manuscripts.

Background: “After head and neck cancer surgery with free flap reconstruction, the use of glucocorticoids is often required to alleviate inflammation and edema. However, the impact of glucocorticoid on postoperative complications and cancer progression remains unclear.”

Conclusion: “The use of high-dose postoperative glucocorticoids is associated with increased postoperative complications, higher readmission rates, and poorer oncological outcomes in patients. The results advocate for cautious use and dosage management of perioperative glucocorticoids in head and neck surgeries to optimize patient outcomes.”

(The position of the original manuscript: Page 2-3, Lines 17-20, 38-43)
7. The aim is clear
8. The KEYWORDS are suitable
9. Lack of the graphical abstract
**Response:**

Thank you for your comment. We highly value your feedback and have taken it into consideration. However, as our article primarily presents data in tabular format, the effectiveness of graphical abstracts is limited. Therefore, we did not provide a graphical abstract.

10. Lack of the abbreviations section

**Response:**

Thank you for your comment. abbreviations sections are present in the manuscript, such as head and neck cancer (HNC), glucocorticoids (GCs). Thanks to your reminder, we have further generalized the abbreviations in the manuscript to make the article more coherent.
11. Lack of the highlight points
12. Arrangement of headings and subheadings are good
13. The introduction provides sufficient background information for readers in the immediate field to understand the problem/hypotheses
14. The paper arrangement is good
15. The method section is clear
16. The depth of the academic material is good
17. The study design is suitable
18. The suitability and accuracy of questions is good
19. The research methodology is reproducible
20. The materials are suitable
21. The inclusion criteria are good
22. The exclusion criteria are logical
23. The logic is clear
24. The presentation of the methods is good
25. Please submit a video abstract if it is available. It is an optional requirement

**Response:**

Thank you for your comment. Sorry, but a video abstract is not available.
26. The paper is not novel. If it is novel, please clarifies how?

**Response:**

Thank you for your kind comments. We truly appreciate your insightful feedback. We believe our study presents several novel aspects:

1. **First exploration clinically of the effect of** **glucocorticoids on long-term postoperative complications (including tumor recurrence and metastasis) in patients undergoing head and neck cancer surgery with free flap reconstruction**:

Although glucocorticoids have traditional applications in anti-inflammatory and immunosuppressive therapy, their role in head and neck cancer patients remains underexplored. Glucocorticoids may influence tumor biology through various mechanisms, including promoting tumor progression and resistance to treatment[1-3]. However, most related research has focused on subclinical studies, with a lack of clinical investigations. Specifically, current clinical studies mainly examine the effects of glucocorticoids on immediate postoperative outcomes and short-term mortality[4-6], with limited research on their long-term impact on tumor recurrence, metastasis, and other factors over extended periods. This study aims to be the first to clinically investigate the long-term effects of exogenous glucocorticoids in the postoperative phase of head and neck cancer patients, including their impact on unplanned readmissions within one year and on tumor recurrence and metastasis after head and neck cancer surgery.

2. **Extensive investigation of postoperative complications**:

Research on the impact of glucocorticoids on postoperative complications in head and neck cancer surgery is currently limited, with a lack of comprehensive clinical studies. This study broadly explores the potential effects of glucocorticoids on postoperative complications (including pulmonary issues, flap complications, and blood glucose control) in patients undergoing head and neck cancer surgery with free flap reconstruction. This approach goes beyond focusing solely on wound healing, blood glucose control, or short-term mortality[4-6]. Additionally, we further investigate the long-term impact of glucocorticoids on postoperative complications, such as tumor metastasis and recurrence, enriching and expanding upon previous studies.

3. **Potential impact of glucocorticoids on postoperative recovery**:

This study focuses on the long-term effects of glucocorticoids on the postoperative recovery of head and neck cancer patients. Not only does this contribute to optimizing postoperative treatment strategies, but it also offers new insights for personalized care plans, potentially improving overall patient prognosis.

In summary, the innovative aspects of this manuscript lie in its systematic investigation of the role of glucocorticoids in postoperative head and neck cancer patients, particularly regarding their impact on postoperative complications, long-term prognosis (including cancer recurrence and metastasis), and postoperative recovery. This research provides a theoretical foundation for personalized treatment and postoperative management.

27. There are few grammatical errors in this article
28. The related concepts are introduced
29. The readability is sufficient
30. The results are interesting
31. The presentation of the results is good
32. The number of tables are adequate
33. Lack of figures?!
34. The structure of the tables are good
35. All tables are well referred to in the text
36. The theoretical analysis in this article is sufficient
37. The discussion of results from multiple angles is sufficient
38. The presentation of the discussion is good
39. The limitation of the study is clear
40. The conclusion is tenable
41. The recommendations are logical
42. The reference section contains many old ref
**Response:**

Thank you for your comment. In recent years, subclinical studies on the relationship between glucocorticoids and head and neck cancers have received extensive attention, so the literature we cited mainly focuses on studies published in the past decade to ensure the cutting-edge background of the research topic. However, authoritative articles and consensus on free flap reconstruction in head and neck cancer surgery have been established as early as the beginning of the 21st century. Therefore, the fact that we cite earlier literature in this regard does not mean that our content lacks novelty.
43. Please use (google scholar and Refseek) search engines then set it since 2020
44. The references are in order within the text
45. References are in one style
46. Bias is present
47. There is no conflict of interest with the author about this topic
48. Fund is mentioned
49. Conflict of interest is mentioned
50. Acknowledgement is mentioned
51. The significance of the paper is good

You can use my suggestions

My final decision is a minor revision

Reviewer #2: Hello. This is a good article and it explores an important topic. Please point out the limitations of the study, such as possible confounding factors and the lack of some information or the lack of accurate data recording in some cases. Also, make sure to mention any suggestions for future studies.

**Response:**

Thank you for your comment. We appreciate your input. Considering your feedback, we have made the necessary revisions of “Limitations” to improve detail. Please refer to the following passage on Page 19-20, Lines 289-305, in the revised version of the manuscript: “This study has several limitations. Primarily, its retrospective design and single-center setting may limit the generalizability of the results. Fortunately, the database we used took effective measures to ensure data quality, and the sample size of our study was relatively large. Secondly, the inherent biases associated with retrospective analyses and the potential presence of unaccounted confounding variables may have affected outcomes. In addition, this study focused solely on unplanned readmissions within 1 year, excluding longer-term complications, which may have led to an underestimation of the true data and restricted our ability to assess the long-term effects and sustainability of the observed outcomes. Longitudinal studies with extended follow-up periods would provide more insight into the persistence of these effects over time. Future research should address these limitations by incorporating more diverse samples, improving data collection precision, and accounting for additional confounding variables. A larger sample size and multicenter designs would enhance the generalizability and reliability of the findings. Furthermore, prospective studies are needed to better define the causal relationships and to explore the mechanisms behind these observations.”

(The position of the original manuscript: Page 20, Lines 297-306).

We appreciate your keen observation and your help in improving the accuracy of our manuscript. We hope that the above answers will respond to your comments and satisfy you. If further adjustments are required or if additional details are necessary, we are open to making the necessary changes.

1.	Cirillo N, Morgan DJ, Pedicillo MC, Celentano A, Lo Muzio L, McCullough MJ, et al. Characterisation of the cancer-associated glucocorticoid system: key role of 11β-hydroxysteroid dehydrogenase type 2. Br J Cancer. 2017;117(7):984-93. Epub 2017/08/11. doi: 10.1038/bjc.2017.243. PubMed PMID: 28797028; PubMed Central PMCID: PMCPMC5625663.

2.	Yang H, Xia L, Chen J, Zhang S, Martin V, Li Q, et al. Stress-glucocorticoid-TSC22D3 axis compromises therapy-induced antitumor immunity. Nat Med. 2019;25(9):1428-41. Epub 2019/09/11. doi: 10.1038/s41591-019-0566-4. PubMed PMID: 31501614.

3.	Bernabé DG, Tamae AC, Miyahara GI, Sundefeld ML, Oliveira SP, Biasoli É R. Increased plasma and salivary cortisol levels in patients with oral cancer and their association with clinical stage. J Clin Pathol. 2012;65(10):934-9. Epub 2012/06/27. doi: 10.1136/jclinpath-2012-200695. PubMed PMID: 22734006.

4.	Kainulainen S, Törnwall J, Koivusalo AM, Suominen AL, Lassus P. Dexamethasone in head and neck cancer patients with microvascular reconstruction: No benefit, more complications. Oral Oncology. 2017;65:45-50. doi: 10.1016/j.oraloncology.2016.12.008.

5.	Kainulainen S, Aro K, Koivusalo AM, Wilkman T, Roine RP, Aronen P, et al. Perioperative Dexamethasone Is Associated With Higher Short-Term Mortality in Reconstructive Head and Neck Cancer Surgery. J Oral Maxillofac Surg. 2020;78(10):1835-45. Epub 2020/06/17. doi: 10.1016/j.joms.2020.05.004. PubMed PMID: 32540321.

6.	Offodile AC, 2nd, Chou HY, Lin JA, Loh CYY, Chang KP, Aycart MA, et al. Hyperglycemia and risk of adverse outcomes following microvascular reconstruction of oncologic head and neck defects. Oral Oncol. 2018;79:15-9. Epub 2018/03/31. doi: 10.1016/j.oraloncology.2018.02.009. PubMed PMID: 29598945.
